# Comparison of Clinical Features and Personality Dimensions between Patients with Major Depressive Disorder and Normal Control

**DOI:** 10.4306/pi.2009.6.3.150

**Published:** 2009-08-03

**Authors:** Ji-Won Hur, Yong-Ku Kim

**Affiliations:** 1 Department of Psychiatry, Korea University College of Medicine, Ansan Hospital, Ansan, Korea.; 2 Division of Brain Korea 21 Biomedical Science, Korea University College of Medicine, Ansan Hospital, Ansan, Korea.

**Keywords:** Major depressive disorder, Temperament and Character Inventory, Aggression, Hopelessness, Impulsiveness

## Abstract

**Objective:**

Personality dimension is considered as a risk factor of depression. This study was to compare aggression, impulsivity, hopelessness, and TCI (temperament and character dimensions) between patients with major depressive disorder (MDD) and normal controls.

**Methods:**

A total of 56 MDD patients and the same number of normal controls who were matched for age, gender, and education were recruited. All subjects completed the following questionnaires; Aggression Questionnaire (AQ), Beck Hopelessness Scale (BHS), Barratt Impulsiveness Scale, 11th Version (BIS-11), and Temperament and Character Inventory (TCI).

**Results:**

MDD patients were significantly higher scores in anger, hostility of AQ, BHS, motor impulsivity of BIS-11, and Harm Avoidances (HA) of TCI with all subscales of HA than normal controls, whereas novelty seeking 1 (NS1) (Exploratory of NS), Reward Dependence (RD) with RD3 (Attachment) · RD4 (Dependence), Self-Directedness (SD) with most subscales of SD, Cooperativeness (CO), and ST3 (Spiritual Acceptance) showed lower scores than normal controls. Moreover, BHS and HA, BIS and NS showed moderate positive correlation in MDD patients, while BHS and SD, HA and SD were negatively correlated.

**Conclusion:**

The present study showed unique clinical features, especially personality dimensions of patients with MDD. Our results could be applicable to suggest treatment process and to predict one's prognosis for depression in that psychological properties are important for drug compliance and treatment response.

## Introduction

Many psychological models of depression have been frequently discussed to indicate the structure and development of depression, although many traits appear to be unstable and easy to be changed by symptoms.^1^ Because traits and factors of mental disorder are important to decide on therapeutic approaches to the specific disorder, previous studies have tried to trace a variety of clinical features and personality dimensions.

Previous researches have narrowed down and focused on some clinical issues to investigate structure of depression for decades. Aggression has been proposed as one of possible psychological variables which explain the depression.^2^ Fava has also suggested that the 44% of the depressed outpatients reported having anger attacks.^3^ Besides, the relation between impulsivity and depression has been considered as a significant feature in depressed patients.^4^ There have been many studies that tried to explain the relation between impulsive aggression and depression with biological factor.^5^ Hopelessness has been considered an another essential factor related to depression, as a proximal sufficient cause of the symptoms of hopelessness depression.^6^ In other word, as hopeless individuals attribute their own negative life-event to stable, global, and internal courses, they could become depressed.

Personality dimension is also considered as a risk factor of depression.^7^ The studies on Temperament and Character Inventory (TCI) by Cloninger et al.^8^ have proposed the relation between personality and depression. TCI includes seven biosocial factors, comprised of four temperament dimensions and three character dimensions. The four dimensions of temperament are Novelty Seeking (NS), Harm Avoidance (HA), Reward Dependence (RD), and Persistence (P). Temperament is defined as stable and heritable dimensions, which is biologically and genetically determined early in life. These dimensions are known to correlate with dopaminergic, serotonic and noradrergic activity. The three dimensions of character are Self-Directedness (SD), Cooperativeness (CO), and Self-Transcendence (ST). Character means the second domain of personality which is predominantly determined by socialization processes during one's whole life.^8^ As TCI includes many variables which have many of implication and description respectively, various results about relationship with depression have been derived.^9^,^10^

Although previous studies have supported the correlation between some subscales of TCI and psychiatric disorders,^11^-^14^ there has been very a few study regarding personality dimensions and the correlation of clinical features and personality in major depressive disorder (MDD). Furthermore, some studies have limitations related to heterogeneity of demographic data, when the covariance such as age or education are not controlled appropriately.^14^

The aim of our study was to compare aggression, impulsivity, hopelessness and personality dimensions between MDD patients and normal controls while controlling for demographic variances; and to evaluate the correlations between variances. Our hypothesis was that MDD patients would have specific personality dimensions than normal control. We also assumed some relationship with the personality and clinical implications.

## Methods

### Patricipants

Among psychiatric patients newly admitted to the closed wards of the Department of Psychiatry, Korea University Medical Center Ansan Hospital, during January 2006 to January 2008, we recruited 56 major depression patients (22 males and 34 females) who met the Diagnostic and Statistical Manual (DSM-IV) criteria.^15^ All patients were medication-free for at least 4 months and had active symptoms at the time of study enrollment. The psychopathological status of the patients was assessed by a trained physician the Hamilton Depression Rating Scale (HDRS).^16^ Patients with a history of any concomitant physical and psychiatric illness, such as anxiety disorder, substance or alcohol abuse, personality disorder and mental retardation were excluded. Patients were found to have a normal physical state as seen from normal values of blood and urine tests (SGOT, SGPT, hemoglobin, hematocrit, serum electrolytes, blood urea, and creatine). Patients were given a standard set of tests by the Venereal Disease Research Laboratory (VDRL) and had normal electrocardiogram (EKG) and electroencephalogram (EEG). For 2 years, through these inclusive/exclusive procedures, 56 MDD patients were selected among 74 MDD inpatients who performed the TCI and questionnaires.

A total of 56 normal control subjects (16 males and 40 females) were recruited. The normal controls were matched with the patients for gender, age and education. They were recruited through advertisement and screened using the Structured Clinical Interview for DSM-IV Axis I Disorders (SCID-I), non-patient version. They had neither medical/psychiatric illness nor family history of mental illness in first- and second-degree relatives. They received a modest fee for completing the interview.

Table 1 shows the demographic data of both groups. The subjects gave informed consent after the study protocol had been fully explained. This study was approved by the Institutional Review Board of Korea University Ansan Hospital.

### Measures

All participants completed the following questionnaires. Aggression Questionnaire (AQ) is self-report measure which contains four subscales of aggression, including physical aggression, verbal aggression, anger, and hostility.^17^ Beck Hopelessness Scale (BHS) was designed to measure the construct of hopelessness that is related positively with depression.^18^ Barratt Impulsiveness Scale, 11th Version (BIS-11) is a short questionnaire developed to measure impulsiveness as a personality trait.^19^ All participants were also administered TCI, which is a self-reported 240 item forced-choice questionnaire.^8^ TCI measures temperament dimensions of NS, HA, RD, P, and character dimensions of SD, CO, ST.

### Statistical analysis

Socio-demographic variables such as age and sex, and clinical history were compared between groups using independent t-tests and χ^2^ tests. Because there were only two groups, the t-test was performed to tap the differences in psychological measures between patients with MDD and normal controls, also. All statistical analyses were done using Statistical Package for Social Science (SPSS) Version 12.0. The level of significance was p<0.05.

## Results

### Comparison of the psychological measures

The mean scores for each measure for both MDD group and normal control are presented in Table 2. MDD group were more like to experience anger (of AQ; 14.63±4.63 vs. 11.77±3.50; t=-3.34, p=0.001), hostility (of AQ; 19.40±6.64 vs. 15.20±5.89; t=-3.07, p<0.01), hopelessness (BHS 9.51±6.77 vs. 2.52±3.15; t=-5.74, p<0.001), and impulsivity tendency (BIS total 51.57±9.95 vs. 47.27±9.88; t=-2.02, p<0.05) including motor impulsivity (BIS; 15.51±4.27 vs. 13.29±3.27; t=-2.81, p<0.01) than normal controls. MDD group also scored significantly higher on all subscales of HA, Worry, pessimism (HA1), Fear of uncertainty (HA2), Shyness (HA3), and Fatigability (HA4). Besides, Exploratory excitability (NS1), Two of RD subscales, Attachment (RD3) and Dependence (RD4), three of SD subscales, Responsibility (SD1), Purposefulness (SD2), Resourcefulness (SD3), and Enlightened second nature (SD5) were lower among MDD patients than controls. CO dimension (C) including Acceptance (C1), Empathy (C2), Helpfulness (C3), and Integrated conscience (C5) subscales and the subscale Spiritual acceptance (ST3) of ST were significantly lower in MDD patients.

### Correlations among scales in major depressive disorder patients

In MDD patients, the correlation between AQ score and ST (r=0.338, p<0.05), BHS score and HA (r=0.548, p<0.01), BIS score and NS (r=0.518, p<0.01), RD and CO (r=0.431, p<0.01), P and SD (r=0.270, p<0.05), SD and CO (r=0.438, p<0.01) were positive (Table 3). Other variables show negative correlations; AQ total score and SD (r=-0.372, p<0.05), AQ score and CO (r=-0.394, p<0.05), BHS score and SD (r=-0.598, p<0.01), BHS score and CO (r=-0.336, p<0.05), BIS score and P (r=-0.364, p<0.05), HA and SD (r=-0.555, p<0.01), and HA and CO (r=-0.450, p<0.01).

## Discussion

The aim of the present study was to explore the psychological properties of MDD compared with normal controls and to tap the risk factor or vulnerability on clinical status and personality dimensions. The major implications of our study are as follows.

The first major finding of this study is that anger, hostility, hopelessness, and impulsivity including motor impulsivity were significant clinical traits in MDD as previous authors have described; Anger and hostility, which were two of subscales of AQ, were higher in MDD patients than normal control (NC) subjects. Pasquini and his colleagues commented also that anger and hostility as well as irritability and aggressiveness are frequent present in depressive patients.^20^ In addition, as previous studied suggested the relationship of hopelessness and depression, similar results was presented in this study also.^6^,^21^ One of the most interesting thing in our study is probably higher impulsivity including motor impulsivity in MDD patients than NC. In fact, Fava et al.^22^ mentioned important issues concerning the impact of MDD on violent and aggressive behavior. Also, there was a notable opinion that activation dimension in depression disorders deserves greater clinical recognition and research.^23^ Similarly, Arciero and Guidano observed prominent symptoms of activation which might be related to depressed patient's cognitive and emotional factors.^24^ Considering these points, it is understandable that MDD patients have rather activation factor, impulsivity including motor impulsivity than NC.

The second major finding of this study is that MDD patients showed higher harm-avoidance, lower RD, SD, and CO in TCI compared to normal controls. These results concur with previous studies that reported positive relation with HA and negative correlation with SD, CO in depressed groups.^9^,^11^,^13^,^25^ HA is the result of depression, and may reflect neurobiological changes in the depressive patients.^7^ Moreover, considering that high score on HA may be better interpreted as reflecting a state rather than a trait, high HA in the "inpatients with MDD" could be understood. On the other hand, MDD patients scored significantly lower RD than normal controls. RD could influence over resilience against psychosocial stressors which results in increased cortisol secretion, subordinance, depressive or suicidal ideation and low self-esteem.^12^ Besides, tendency of SD, which is correlated with accepting responsibility, long-term goals and self-esteem,^26^ was lower in depressed patients than normal controls. Above features of SD could be useful reasons to explain personality of MDD patients in regard to their feelings of futility about future and low self-esteem. Also, as above mentioned, lower score on CO was founded in MDD groups as previous researcher pointed out.^27^ These findings might indicate malfunctioning occurrence pattern. Some authors have proposed yet that CO as well as SD is a dimension which reflects a general psychopathology.^9^ Focusing the concept of CO-socially intolerant, unhelpful, and revengeful-, it seems that this personality dimension makes patient's social adjustment be difficult.

In our study, MDD patients showed significant low exploratory excitability score among NS subscales, also. Even though many researchers pointed insignificant score on subscales NS,^28^,^29^ Hansenne et al.^9^ have reported the results same as ours, interestingly. They explained that the result could be observed in that low exploratory excitability means not doing initiative novel behaviors and producing active exploration of the environment. Finally, spiritual acceptance of ST, which was identified as being important to older adult's sense of well-being,^30^ is lower in MDD group than normal. In the view of biological model for this illness, this result is reasonable. Since correlation between spiritual acceptance and 5-HT1A genotype was defined^31^ which response in unipolar depression.^32^

First, BHS (hopelessness) and HA, BIS (Impulsivity) and NS showed moderate positive Pearson correlation coefficients in MDD patients. The former results is similar with a previous study that suggested the biological effects of relationship between hopelessness and avoidance behavior.^12^ In the other hand, although we didn't describe about correlations in normal controls above, the same results with latter one was found among normal control in case of BIS and NS (r=0.586, p<0.01). Hence, it is considered this relationship about BIS and NS is not due to the characteristic trait of depression.

Secondly, BHS (hopelessness) and SD, HA and SD were negatively correlated. Cloninger et al.^8^ have suggested individual with low level in SD to be weak, fragile, ineffective, irresponsible, and poorly integrated when they are not performing to the direction of a mature leader. These features could indicate negative correlations between SD and hopelessness, HA. In addition, Chien and Dunner^29^ and other researchers^25^ have described that reductions in depressive symptoms was accompanied by decreases in HA and increases in SD longitudinally same as our results.

In this study, we explored the clinical features, temperament and characteristics of patients with MDD as a cue factor. Sato and Uehara have asserted the study of personality as a precursor of depression helped us to understand treatment and etiology.^33^ Since most studies didn't cover symptoms of 'activation' such as anger, irritability, aggressiveness, and hostility among depressed patients,^23^ our results about not only TCI but also AQ, BIS and BHS could be a references for further studies. Another strong point of our study is that, when the analysis was administered, statistical traits such as sex and education which could carry unwanted effects were controlled for homogeneity of demographic data.

Our study is limited by sampling and cross-sectional design. Since only inpatients with MDD but not outpatient were sampled, there might be bias by severity of illness. Besides, we didn't perform longitudinal study for following the phase of above psychological properties after discharge. Finally, we didn't classified as either endogenic or exogenic depression which have different processes of onset, procedure and prognosis. Further studies are necessary to conclude that these results could be maintained with refined and classified patients in the various courses of depression.

Our results suggest that MDD patients have significant clinical features and personality dimensions, which were related partly. The results included activation dimensions as anger, hostility and impulsivity as well as personality dimensions. Further longitudinal study is necessary to confirm whether the characteristics of MDD patients are state or trait.

## Figures and Tables

**TABLE 1:**
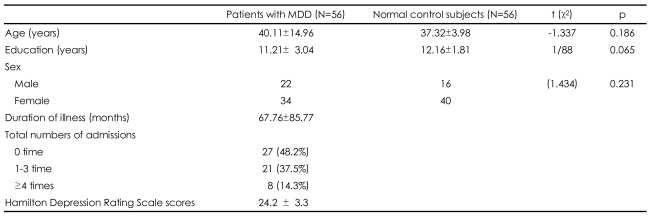
Demographic data for patients with major depressive disorder (MDD) and normal control subjects

mean±SD

**TABLE 2:**
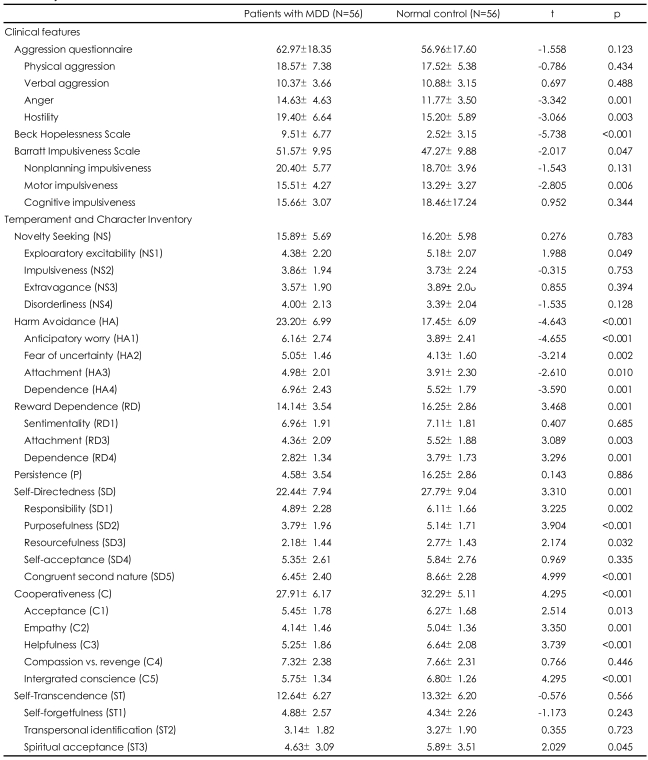
Comparison of clinical features and personality dimensions between patients with major depressive disorder (MDD) and normal control subjects

mean±SD

**TABLE 3:**
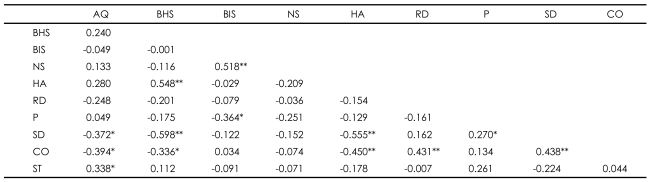
Correlations among clinical features and personality dimensions within the Major depressive disorder group

^*^p<0.05, ^**^p<0.01. AQ: Aggression Questionnaire, BHS: Beck Hopelessness Scale, BIS: Barratt Impulsiveness Scale, NS: Novelty Seeking, HA: Harm Avoidance, RD: Reward Dependence, P: Persistence, SD: Self-Directedness, CO: Cooperativeness, ST: Self-Transcendence
